# Validity and reliability of the Greek version of Wijma delivery expectancy/experience questionnaire (Version A) among low-risk pregnant women

**DOI:** 10.1186/s40359-024-01662-4

**Published:** 2024-03-19

**Authors:** Pinelopi Varela, Ioannis Zervas, Victoria Vivilaki, Aikaterini Lykeridou, Anna Deltsidou

**Affiliations:** 1 Department of Midwifery, University of West Attica, Athens, Greece; 2 National and Kapodistrian University of Athens Medical School, Eginition University Hospital, Athens, MD Greece

**Keywords:** Fear of childbirth, Wijma Delivery Expectancy/Experience Questionnaire, Psychometric properties, Validity, Reliability, Exploratory factor analysis, Confirmatory factor analysis, Greece

## Abstract

**Background:**

Fear of childbirth is a frequent health issue for pregnant women. The Wijma Delivery Expectancy/Experience Questionnaire (W-DEQ) is a widely used instrument to measure the fear of childbirth during the antenatal period. The aim of the study was to assess the psychometric properties of the W-DEQ (version A) in a sample of Greek pregnant women.

**Methods:**

Low-risk pregnant women in the second or third trimester of pregnancy (*N* = 201) were invited to participate in the study and to complete a booklet of questionnaires including the Greek versions of W-DEQ-A, State-Trait Anxiety Inventory (STAI), Coping Orientations to Problems Experienced (Brief COPE), Perceived Stress Scale (PSS-10) and Edinburgh Postnatal Depression Scale (EPDS). Exploratory (EFA) and confirmatory factor analysis (CFA) was performed.

**Results:**

The mean age of participants was 34.2 years (SD = 4.3 years). EFA yielded six factors (“Lack of self-efficacy”, “Lack of positive anticipation”, “Lack of feeling lonely”, “Concerns about delivery and losing control”, “Calmness”, and “Concern for the child”) of 33 items of W-DEQ-A. CFA confirmed the multidimensionality of the instrument. All Cronbach’s alpha were over 0.7, indicating acceptable reliability of the factors. All factors were significantly correlated with each other, and convergent validity was demonstrated by a significant association with stress, anxiety, and depression among low-risk pregnant women.

**Conclusion:**

The Greek version of W-DEQ-A proved to be a valid and reliable instrument of fear of childbirth among Greek low-risk pregnant women.

**Supplementary Information:**

The online version contains supplementary material available at 10.1186/s40359-024-01662-4.

## Background

“If they are primiparous, the expectation of unknown pain preoccupies them beyond all measure, and throws them into a state of inexpressible anxiety. If they are already mothers, they are terrified of the memory of the past and the prospect of the future” was the description by the French psychiatrist Louis Victor Marcé (1858) regarding the fear of childbirth (FOC) [1]. Since then, during the twentieth and twenty-first centuries, the issue of FOC has received impressive scientific and clinical attention on a global scale. FOC, which manifests as symptoms including worries or extreme fear, may develop during pregnancy [2, 3]. Pregnant women often worry about the neonate’s safety, their suffering, losing control, getting medical help, and having a labor that goes in an unusual direction [4–6]. Also, they frequently fear for the pain during delivery or have concerns about being able to give birth [7, 8]. Reactions surrounding the fear of the pain during labor and fear of the unknown are thought to be natural for women who are going through pregnancy and the impending birth for the first time. But in spite of that, for some women, their fear of giving birth goes beyond primary worry, leading to tokophobia, a severe fear of pregnancy or childbirth, and experiencing intense anxiety and avoidance of ideas and actions [9, 10].

The prevalence rates of FOC it is noticed that varies across countries. Early research found that 20% of pregnant women in Scandinavia had FOC, with 5–10% of those women reporting severe fear [11]. The rates in European countries range from 1.9 to 14% [12] while studies from Australia indicate higher rates of around 30 − 48% [13, 14]. A systematic review demonstrated that the prevalence of FOC in nine countries in Europe, Australia, Canada, and the United States varied from 6.3 to 14.8% [15]. Despite the variations in the percentages that were seen, research findings so far have demonstrated that FOC has been linked to a number of unfavorable outcomes. Adverse consequences include the prolonged period of labor [16, 17] the use of anesthesia during labor [17, 18], the manifestation of obstetric complications [17], and the increased risk of cesarean section (CS) [19, 20]. Aside from the above, the presence of traumatic stress symptoms [17, 21], the negative birth experience [2], the emotional imbalance, the need for psychiatric care and the increased risk of postpartum depression and, as a result, an impaired maternal–infant relationship [4, 17, 22] are among the reported outcomes in women with FOC. Research on the mental health of women in Greece following childbirth has revealed that Greek women experience poor mental health in the postpartum period, with rates of postpartum depressive symptoms ranging from 12.1 to 59.6% [23–25]. These symptoms were found to be significantly correlated with stressful events during pregnancy and to develop significantly more frequently in mothers who gave birth by caesarean section as opposed to vaginal delivery [23, 25].

For the assessment and measurement of the FOC, a variety of measurement tools and methods have been developed [15], but the most widely employed used instrument for evaluation is the Wijma Delivery Expectancy/Experience Questionnaire (W-DEQ) [15, 26]. The W-DEQ was developed in Sweden [27], and in line with Lazarus’ theory, which argues that people’s response to environmental stressors is largely determined by their appraisal processes [27, 28]. There are two versions of this instrument; W-DEQ version A can be administrated before birth to assess expectations and the W-DEQ version B can be administrated after birth to assess the women’s experiences. Both versions are appropriate for assessing FOC in both nulliparous and multiparous women. There are 33 items in each version, and responses are graded on a six-point Likert scale, from “not at all” to “extremely”. Item scores are added to obtain a total score. The lowest score allowed is zero, and the highest allowed is 165. Scores for items 2, 3, 6, 7, 8,11, 12, 15, 19, 20, 24, 25, 27, and 31 should be reversed. The higher the score is, the greater the fear of childbirth is demonstrated [27]. The internal consistency of original version A is excellent (Cronbach’s alpha = 0.93). The validity of version A was confirmed by correlations with the following instruments; the Fear Questionnaire (FQ) [29], the S-R Inventory of Anxiousness (SRI) [30], the State-Trait Anxiety Inventory (STAI) [31], the Karolinska Scales of Personality (KSP) [32], the Eysenck Personality Inventory (EPI) [33], the Internal-External Locus of Control Scale (I-E) [34], and the Beck Depression Inventory (BDI) [35]. The findings demonstrated that correlations between the W-DEQ, FQ, and SRI were of the similar level as those with tools used to measure general anxiety. Also, it was confirmed that the correlation of the W-DEQ with the other questionnaires was the same for both the nulliparous and multiparous women, except for FQ-childbirth, because for the multiparous group this correlation was stronger (FQ-childbirth (*r* =.78) and statistically significant (p-value = 0.0001). Furthermore, the strongest finding from scale intercorrelations showed a correlation between the W-DEQ and STAI. In the multiparous group the W-DEQ correlated with the SRI and with the FQ-childbirth demonstrating that the W-DEQ and these questionnaires had a greater shared variance than the W-DEQ and questionnaires assessing general anxiety. In other words, the W-DEQ also measures aspects related to direct communication of fear of childbirth (FQ-childbirth) and physiological symptoms related to fear when imagining labor and delivery (SRI) [27]. Even though it was first developed as a unidimensional scale [27] subsequent factor analysis research on several populations has validated its multifactorial structure [36–39].

In Greece, the study of FOC is not extensive, and there is limited available data. Since there are scientific data on the adverse effects of FOC and ongoing research on the factor analysis of W-DEQ, the Greek validation of the W-DEQ version A appears reasonable and essential for both scientific and research reasons. Therefore, the aim of this study was to assess the psychometric characteristics of W-DEQ version A (i.e., confirmatory factor analysis, exploratory factor analysis, concurrent validity, and internal consistency reliability) among nulliparous and multiparous Greek women. The hypothesis of the present study was that W-DEQ-A is a valid and reliable instrument and, moreover, is correlated with other scales of depression, stress, anxiety, and coping strategies.

## Methods

### Translation and pilot test

After receiving the approval of the author who developed the scale (Professor Klaas Wijma), the translation process was initiated. The process included four stages; forward translation, synthesis of the translations, back translation and Expert Committee and submission of documentation to the developer. The test–retest reliability of the scale came up after the administration of the instrument to the same sample group of 30 pregnant women at different times. Ten days was the time interval between the two administrations. The test–retest reliability (intraclass correlation coefficients, ICC) for W-DEQ version A ranged from 0.88 to 1.00 and Cronbach’s a reliability coefficient was 0.91. The detailed information about the translation process and the pilot study have been published [40]. After the completion of the pilot study, the Greek version of the W-DEQ (GrW-DEQ) version A was developed.

### Participants and procedure

Pregnant women throughout their second or third trimester of pregnancy were invited to participate in the study during their routine antenatal examination by the principal researcher. The study was conducted in a public maternity hospital in Athens from July 2020 to July 2021. The inclusion criteria were as follows: low-risk pregnant women aged over 18 years, with an adequate understanding of the Greek language. Pregnant women having a severe chronic disease, a high-risk pregnancy, a psychiatric illness, or intaking psychiatric medication, having twin or multiple pregnancies, were excluded. Two hundred one of the 240 invited women accepted the invitation to participate and signing an informed consent form. The participants were given a questionnaire booklet, which they were informed to submit at their subsequent follow-up appointment. Six self-administrated questionnaires were included in the booklet from which the first of them was a questionnaire with demographic characteristics and questions concerning mental health and obstetric history. The rest questionnaires are described below.

### Measures

The Wijma Delivery Expectancy/Experience Questionnaire version A (W-DEQ-A). The W-DEQ-A is a self-report measure of FOC, with its psychometric properties showing a valid and reliable tool [27]. Participants were asked to fill in the Greek version of the W-DEQ-A (GrW-DEQ-A).

The State-Trait Anxiety Inventory (STAI): There are two subscales that comprise the STAI. Anxiety at the moment of assessment, which might change over time, is measured by the State subscale. The Trait subscale evaluates anxiety level as a persistent personal feature, which is stable over time. Each subscale consists of 20 items, each of which is graded from 1 to 4 on a Likert scale. The total score ranges from 20 to 80, for each subscale, and higher scores indicate higher levels of anxiety.

 [41]. The questionnaire has been translated and validated in the Greek population and Cronbach’s alpha was found to be 0.93 for the state anxiety subscale and 0.92 for the trait anxiety subscale [42].

Edinburgh Postpartum Depression Scale (EPDS): EPDS is a valid and reliable tool used both in the prenatal and postnatal populations. Ten statements describing depressive symptoms comprise up the scale, which has four alternative responses, each of which is graded according to how severe or long-lasting the symptom is. The answers are scored from 0 to 3, and at the end, their total sum is calculated [43]. The EPDS scale has been translated and validated in the Greek population, with the internal consistency reliability of the scale being excellent (Cronbach’s alpha = 0.9) [44].

Coping Orientations to Problems Experienced (Brief COPE). The COPE evaluates dispositional or situation-specific coping by assessing a number of different coping strategies used by people in general or in a specific scenario. The Brief COPE is a 28-item measure of individuals’ strategies for coping with problems and stress. Fourteen coping strategies are measured by the items, which respondents’ rate on a four-point Likert scale ranging from “not at all” to “very much” [45]. The Brief COPE scale has been translated and validated in the Greek population, with sufficient psychometric characteristics. Cronbach’s alpha of the 14 two-item scales ranged from 0.48 to 0.93 [46].

The Perceived Stress Scale (PSS-10). The 10-item PSS scale measures how stressful experiences are perceived by asking the respondent to score the frequency of their feelings and thoughts in relation to incidents and circumstances that occurred during the preceding month. On a five-point Likert scale (0 = never to 4 = very often), each item is scored. Total scores are calculated after reversing positive items’ scores and then summing up all scores. Total scores for PSS-10 range from 0 to 40. A higher score indicates greater stress [47]. The Greek version of the PSS-10 presented satisfactory psychometric properties and a Cronbach’s alpha of 0.82 [48].

### Statistical analysis

Quantitative variables were expressed as mean values (Standard Deviation) and as median (interquantile range), while qualitative variables were expressed as absolute and relative frequencies. Confirmatory factor analysis (CFA), with maximum likelihood estimation method, was conducted in order to test how well the W-DEQ-A one-factor model fits the data. We used the chi-square by degrees of freedom ratio (χ2/df), the comparative fit index (CFI), the Tucker Lewis index (TLI), the root mean square error of approximation (RMSEA) and the standardized root mean square residual (SRMR) as goodness-of-fit indices [49], and these parameters were considered adequate when χ2/df ≤ 2.0, CFI ≥ 0.90, TLI ≥ 0.90 RMSEA ≤ 0.05 and SRMR < 0.08 [50–53]. To compare the different W-DEQ-A versions, we computed Akaike information criterion (AIC). Exploratory factor analysis (EFA) was conducted to assess the construct validity of W-DEQ-A scale. The adequacy of the data was confirmed using the Kaiser-Meyer-Olkin (KMO) method with > 0.6 considered acceptable, and a significant Bartlett’s test of sphericity. Principal component analysis and varimax rotation were used to extract factors and improve the interpretability of the solution. The number of factors retained was based on an eigenvalue of > 1 and an assessment of the scree plot. A factor loading of ≥ 0.40 was used to identify whether an item satisfactorily represented its factor. Spearman’s correlation coefficient was used to assess the concurrent validity. We tested the extent to which the W-DEQ-A scale and its factors were correlated with EPDS, PSS, Brief-Cope and STAI scales. Internal consistency reliability was determined by the calculation of Cronbach’s alpha coefficient. Scales with reliabilities equal to or greater than 0.70 were considered acceptable. All reported p values are two-tailed. Statistical significance was set at *p* <.05 and analyses were conducted using SPSS statistical software (version 22.0).

## Results

### Sociodemographic characteristics

The sample consisted of 201 pregnant women, with mean age 34.2 years (SD = 4.3 years). The most women were Greek (96.0%) and married or living with their partner (96.5%) (Table 1). University graduates was 60.7% of the sample and 59.7% were working full-time. In addition, 52.7% of the women had monthly family income 1,000–3,000 euro and 48.8% had children. Also, 29.9% had visited a specialist for psychological problems in the past and 5.5% had taken treatment for psychological reasons. Psychotherapy had done 22.4% of the sample and 40.8% had lived a stressful event during last year. Furthermore, 24.4% had been abused during childhood, 23.9% during adulthood and 23.9% during a visit to a health professional (Table 1).

**Table 1:** Sample characteristics

	N (%)
Age, mean (SD)	34.2 (4.3)
Nationality	
Greek	193 (96.0)
Other	8 (4.0)
Married/ Living with partner	194 (96.5)
Educational level	
High school at most	57 (28.4)
University	122 (60.7)
Postgraduate degree	22 (10.9)
Occupation	
Full time employee	120 (59.7)
Part time employee	24 (11.9)
Free-lancer	10 (5.0)
Unemployed	29 (14.4)
Household	18 (9.0)
Monthly family income	
Up to 1.000 €	74 (36.8)
1.000–3.000 €	106 (52.7)
≥ 3.000 €	21 (10.4)
Having children	98 (48.8)
Visited a specialist for psychological problems in the past	60 (29.9)
Ever received treatment for psychological reasons	11 (5.5)
Psychotherapy in the past	45 (22.4)
Being abused during childhood	49 (24.4)
Being abused during adulthood	48 (23.9)
Being abused during a health service visit	48 (23.9)
Stressful event during last year	82 (40.8)
EPDS scale, mean (SD)	5.2 (4.2)
PSS-10, mean (SD)	14.8 (6.4)
Active positive coping, mean (SD)	26.6 (4.9)
Behavioural disengagement, mean (SD)	4.1 (1.4)
Substance abuse, mean (SD)	2.2 (0.7)
Seeking support, mean (SD)	10.9 (3.2)
Religion, mean (SD)	4.2 (1.9)
Humor, mean (SD)	4 (1.4)
Avoidance, mean (SD)	6.9 (2)
Express negative feelings, mean (SD)	7.3 (2.1)
State, mean (SD)	39.0 (10.5)
Trait, mean (SD)	41.4 (7.4)

### Confirmatory and exploratory factor analysis

CFA was conducted in order to check the one-factor solution of the original W-DEQ-A scale and it was found that this solution had a very poor model fit (χ^2^/df = 3.28; RMSEA = 0.11; CFI = 0.62; TLI = 0.60 and SRMR = 0.09). Thus, EFA was conducted in order to investigate the internal structure of W-DEQ-A. The sample adequacy was confirmed by a KMO of 0.89 and a significant Bartlett’s test, *p* <.001. Six factors yielded from EFA with Varimax rotation that accounted for 59.6% of the variance. Their loadings are presented in Table 2, as well as the variance explained by each factor. Factor “Lack of self-efficacy” consisted by 11 items and explained 12.7% of the variance. Factor “Lack of positive anticipation” consisted of 4 items and explained 12.4% of the variance and factor “Lack of feeling lonely” consisted of 7 items and explained 11.7% of the variance. Also, factor “Calmness” consisted of 4 items and explained 8.4% of the variance and factor “Concerns about delivery and losing control” consisted of 5 items and explained 8.4% of the variance. The 6th factor “Concern for the child” consisted of 2 items and explained 6% of the variance (Table 2). CFA was conducted in order to check the new six-factor solution of the W-DEQ-A scale and it was found that this solution had an adequate model fit (χ2/df = 1.75; RMSEA = 0.03; CFI = 0.93; TLI = 0.91 and SRMR = 0.06). According to AIC, it can be concluded that the 6-item version had lower value compared to the one-factor version (17,823.11 vs. 18,329.73), indicating that it was better.

**Table 2:** Factor loadings from EFA and percentages of variance explained

Items	Lack of self-efficacy	Lack of positive anticipation	Lack of feeling lonely	Concerns about delivery and losing control	Calmness	Concern for the child
4	0.62					
5	0.67					
6	− 0.44					
9	0.55					
10	0.63					
13	0.50					
14	0.48					
18	0.43					
23	0.55					
17	0.50					
22	0.60					
21		0.71				
28		0.71				
29		0.64				
30		0.64				
3			0.77			
7			0.61			
8			0.53			
11			0.70			
15			0.60			
20			0.64			
31			0.46			
1				0.43		
2				− 0.55		
25				− 0.58		
26				0.51		
27				− 0.57		
16					− 0.49	
12					0.56	
19					0.61	
24					0.75	
32						0.91
33						0.88
% Variance explained	12,7	12,4	11,7	8,4	8,4	6.0

### Internal consistency

The reliability of each factor is presented in Table 3. All Cronbach’s alpha were over 0.7, indicating acceptable reliability of the factors. Moreover, when one item of one factor was removed, trivial change was made in its alpha, thus no item needed to be removed.

**Table 3:** Item-total correlations and Cronbach’s a of W-DEQ-A items

Factor	Item	Corrected Item-Total Correlation	Cronbach’s Alpha if Item Deleted	Cronbach’s Alpha
Lack of self-efficacy	4	0,65	0,88	0,89
	5	0,74	0,88	
	6	0,51	0,89	
	9	0,61	0,88	
	10	0,57	0,89	
	13	0,65	0,88	
	14	0,63	0,88	
	17	0,54	0,89	
	18	0,63	0,88	
	22	0,74	0,88	
	23	0,60	0,89	
Lack of positive anticipation	21	0,44	0,80	0,79
	28	0,58	0,74	
	29	0,70	0,67	
	30	0,67	0,69	
Lack of feeling lonely	3	0,60	0,80	0,83
	7	0,60	0,80	
	8	0,59	0,81	
	11	0,71	0,79	
	15	0,52	0,82	
	20	0,68	0,79	
	31	0,34	0,84	
Concerns about delivery and losing control	1	0,44	0,65	0,70
	26	0,35	0,69	
	2	0,45	0,64	
	25	0,49	0,63	
	27	0,53	0,61	
Calmness	16	0,52	0,58	0,70
	12	0,36	0,68	
	19	0,59	0,53	
	24	0,39	0,66	
Concern for the child	32	0,79	-	0,88
	33	0,79	-	

### Correlation coefficients between W-DEQ-A factors

Descriptives of each factor as well as the correlation coefficients between them are presented in Table 4. All factors were significantly correlated with each other, in a way that greater concerns were associated with greater lack of self-efficacy, positive anticipation, feeling lonely and less calmness. Also, greater lack of self-efficacy, positive anticipation, feeling lonely was associated with less calmness (Table 4).

**Table 4:** Descriptive statistics for W-DEQ-A factors and their correlations between them

		Minimum	Maximum	Mean (SD)	Median (IQR)	Spearman’s rho coefficients					
						1	2	3	4	5	6
1	Lack of self-efficacy	0.00	3.91	1.93 (0.82)	1.91 (1.36 ─ 2.45)	1	0.59***	− 0.64***	0.59***	− 0.45***	0.33***
2	Lack of positive anticipation	0.00	3.25	0.73 (0.72)	0.5 (0 ─ 1)		1	− 0.46***	0.42***	− 0.22**	0.20***
3	Lack of feeling lonely	1.00	5.00	3.82 (0.87)	3.86 (3.43 ─ 4.57)			1	− 0.64***	0.54***	− 0.31***
4	Concerns about delivery and losing control	0.00	3.40	1.5 (0.72)	1.6 (1 ─ 2)				1	− 0.53***	0.32***
5	Calmness	0.50	4.75	2.5 (0.93)	2.5 (1.75 ─ 3)					1	− 0.21**
6	Concern for the child	0.00	5.00	0.93 (1.18)	0.5 (0 ─ 1.5)						1

*p <.05; **p <.01; ***p <.001

### Convergent validity

Worse feelings about labor and delivery were significantly associated with greater stress, anxiety and depression. Also, worse feelings about labor and delivery were significantly associated with greater use of behavioral disengagement, substance abuse, avoidance and expression of negative feelings. Moreover, greater calmness was significantly associated with more religious way of coping stressful conditions (Table 5).

**Table 5:** Spearman’s correlation coefficients of WDEQA factors with EPDS, PSS, Brief-cope and STAI scales

	Lack of self-efficacy	Lack of positive anticipation	Lack of feeling lonely	Concerns about delivery and losing control	Calmness	Concern for the child
PSS-10	0.38***	0.28***	− 0.39***	0.34***	− 0.32***	0.34***
Active positive coping	− 0.05	− 0.13	0.08	− 0.12	0.09	− 0.10
Behavioral disengagement	0.18**	0.07	− 0.19**	0.16**	− 0.14**	0.10
Substance abuse	0.18*	0.24**	− 0.17*	0.17*	− 0.03	0.06
Seeking support	0.07	− 0.04	− 0.04	− 0.07	− 0.01	0.07
Religion	− 0.02	− 0.04	0.09	− 0.11	0.24**	0.07
Humor	0.05	0.03	− 0.12	0.10	− 0.07	− 0.01
Avoidance	0.11	0.02	− 0.14*	0.18*	− 0.21**	0.08
Express negative feelings	0.26***	0.10	− 0.24***	0.09	− 0.24**	0.08
State	0.47***	0.30***	− 0.50***	0.40***	− 0.34***	0.35***
Trait	0.40***	0.26***	− 0.42***	0.34***	− 0.33***	0.34***
EPDS scale	0.36***	0.27***	− 0.32***	0.34***	− 0.33***	0.32***

*p <.05; **p <.01; ***p <.001

## Discussion

The aim of the present study was to assess the psychometric characteristics of the Greek version of W-DEQ-A. Results of CFA confirmed that the instrument is not a unidimensional measure and EFA confirmed that the Greek W-DEQ version A (GrW-DEQ-A) has a six-factor structure with 33 items. The Greek version of W-DEQ-A (GrW-DEQ-A) also has acceptable internal consistency, all factors were significantly correlated with each other, and convergent validity was demonstrated by a significant association with stress, anxiety, and depression among Greek low-risk pregnant women.

Table 2 indicates that the EFA produced six factors (lack of self-efficacy, lack of positive anticipation, lack of feeling lonely, concerns about delivery and losing control, calmness, and concern for the child). The multidimensionality of the scale identified in the present study is consistent with several other studies from different countries [36–38, 54, 55]. Although the multidimensional structure of the W-DEQ-A is a common finding of previous studies, the number of factors varies across studies. Thus, some studies noticed three factors [36, 56], four factors [37, 55, 57], five factors [58], six factors [38, 39, 59], seven factors [60], and nine factors [58, 61]. The six-factor structure of GrW-DEQ-A is in line with the number of the factor structure of the studies by Lukasse et al. [38], Mortazavi [39] and Andaroon et al. [59] and is consistent with the study of Lukasse et al. [38] regarding the number of the items.

Despite the fact that there are many instances when the factors in our study share the same items as those of other authors, there are also some differences. This observation emphasizes that although there are cultural differences and dissimilarities regarding obstetric and perinatal care, women from country to country share some common factors of FOC.

In the current study, no item had to be removed and six factors yielded from EFA that significantly correlated with each other, and accounted for 59.6% of the variance. This result is consistent with previous studies in Hungary [55] and Iran [59] which reported 60.25% and 58.8% respectively of the variance. Factors “Lack of self-efficacy”, “Lack of positive anticipation” and “Lack of feeling lonely” had the highest impact on total variance (12.7%, 12.4%, and 11.7% respectively). The final six-factor structure of GrW-DEQ-A identified in the present study was ideal and comprehensible. This study may represent cultural beliefs about FOC held by Greek women.

With a Cronbach’s alpha for each of the six factors over 0.7, the Greek version of the W-DEQ-A demonstrated satisfactory internal consistency, indicating acceptable and robust scale reliability. In terms of convergent validity, all factors of GrW-DEQ-A were significantly associated with PSS-10, STAI and EPDS. Also, all factors were significantly associated with strategies for coping and more specifically, greater use of Behavioral disengagement, Substance abuse, Avoidance and Expression of negative feelings. These results show that the scale correlates with other measures of stress, anxiety, depression and coping strategies.

The findings of the present study confirm the good psychometric properties of the Greek version of the W-DEQ-A. The clinical implications relevant to this observation are worth noticing. Health professionals in perinatal care can easily use the Greek version of the W-DEQ-A in clinical practice as an efficient assessment and screening tool for silent concerns and worries in Greek women during pregnancy. Following the women’s comments, having a close-knit conversation with them will reveal the origins of their worries and anxieties. Perinatal care health practitioners might carefully examine changing the circumstances in which women give birth while taking into consideration the information from all of these discussions.

The following limitations must be taken into consideration when evaluating the study’s findings. The results may not be applicable to women with high-risk pregnancies because we only included low-risk pregnant women. The results of this study cannot be generalized to women who do not get regular prenatal care since the women who participated in it were frequent attendees to the prenatal clinics at the study hospital. The study sample came from a large urban center in Greece. Therefore, the results cannot be safely generalized to the rural pregnant population of the country. Perhaps the construction of FOC for pregnant women who live in rural areas is different from that of pregnant women who live in urban centers. Despite these limitations, this is the first study that evaluated the validity and reliability of a Greek version of the W-DEQ-A among Greek low-risk pregnant women. The tool will give healthcare professionals a starting point for understanding the phenomenon of the FOC in Greece. Also, can lead to the further development of interventions that will enhance the standard of healthcare and the outcomes of childbirth for Greek women. Further research, particularly replications of the study in samples of high-risk pregnancies is needed. These future studies will allow us to better explore and understand if the construct of FOC is differentiated to this group of pregnant women. Also, it will allow us to make comparisons with low-risk pregnancies.

## Conclusions

The current study presented a valid and reliable Greek version of W-DEQ-A with the inclusion of 33 items and resulting in six factors, confirming the multidimensionality of the instrument. The Greek version of W-DEQ-A proved to be suitable and is suggested for research and clinical purposes in low-risk pregnant Greek women. Thus, the tool is recommended to be used for measuring fear of childbirth in Greek women.

### Electronic supplementary material

Below is the link to the electronic supplementary material.

Supplementary Material 1

## Data Availability

Upon rational demand, the corresponding author will provide the datasets used and analyzed during the current study.

## Abbreviations

CFA: Confirmatory factor analysis
CFI: Comparative fit index
COPE: Coping Orientations to Problems Experienced
CS: Cesarean section
EFA: Exploratory factor analysis
EPDS: Edinburgh Postnatal Depression Scale
FOC: Fear of childbirth
GrW-DEQ-A: Greek version of W-DEQ-A
KMO: Kaiser-Meyer-Olkin
PSS-10: Perceived Stress Scale
RMSEA: Root mean square error of approximation
SRMR: Standardized root mean square residual
STAI: State-Trait Anxiety Inventory
TLI: Tucker Lewis index
W-DEQ: Wijma Delivery Expectancy/Experience Questionnaire
χ2/df: Chi-square by degrees of freedom ratio
