# Supplementary material for: Validity and reliability of the Greek version of Wijma delivery expectancy/experience questionnaire (Version A) among low-risk pregnant women
Source: BMC Psychol. 2024 Mar 19;12:165. doi: 10.1186/s40359-024-01662-4 (PMC10953171; doi:10.1186/s40359-024-01662-4)
Supplement: Supplementary file 1 — Supplementary Material 1 [file 40359_2024_1662_MOESM1_ESM.pdf]

Psychometric aspects of the W-DEQ; a new questionnaire for the measurement of fear of childbirth

K. Wijma[1], *B. Wijma*[1] *and M. Zar*[2]

[1]Department of Obstetrics and Gynaecology, Linköping University, Linköping; and
[2]University College of Health Sciences, Jönköping, Sweden

Key words: Fear of Childbirth, W-DEQ, Psychometrics, Fear, Anxiety, Delivery

J Psychosom Obstet Gynecol Downloaded from informahealthcare.com by University of Connecticut on 10/11/14
For personal use only.

ABSTRACT

Up to now it has been difficult to study fear of childbirth because of a shortage of adequate psychological measurements. Therefore the Wijma Delivery Expectancy/Experience Questionnaire (W-DEQ) was developed. This paper presents the theoretical background of the W-DEQ together with a documentation of the first psychometric studies. Examination of construct validity indicates that it seems to be possible to penetrate a psychological construct related to fear of childbirth by means of the W-DEQ, both before and after delivery, in nulliparous as well as in parous women. The questionnaire measures the construct more clearly in parous than in nulliparous women. Internal consistency reliability and split-half reliability of the W-DEQ of ≥ 0.87 are good for a new research instrument. More research is on its way to make the W-DEQ suitable even for measurements in applied settings.

INTRODUCTION

Background

As a biological process, the delivery is characterized by a series of distinct and predictive physiological phenomena. Yet for the individual woman the exact course as well as the subjective experience of the anticipated delivery are unknown. A pregnant woman may therefore question her capability to cope with the challenges delivery brings. Feelings of uncertainty and anxiousness may arise from the woman's experience of being captured in a situation where she faces the approaching delivery, which is unknown, uncontrollable and unavoidable. For some women this situation is psychologically so distressing that it generates fear of childbirth.

Reviews[1–4] show that anxiety during pregnancy, so far almost exclusively, has been studied by means of psychological instruments designed to measure anxiety in general. However, it could be thought that fear of childbirth is a psychological domain of its own, and, in that case, instruments for the identification of anxiety in general would be less suitable for adequately measuring this specific fear.

After experiences with the development of another kind of questionnaire for the measurement of fear of childbirth[5], the instrument presented here has been developed from the theory that the expectations a pregnant woman has about the anticipated delivery are highly relevant for both her experience of and behavior during the delivery. Similarly the woman's appraisal of a past delivery will strongly indicate the degree of anxiety associated with her last childbirth, offering an

Correspondence to: Dr K. Wijma, Department of Obstetrics and Gynaecology, Faculty of Health Sciences, Linköping University, S-581 85 Linköping, Sweden

RIGHTSLINK

J Psychosom Obstet Gynaecol Downloaded from informahealthcare.com by University of Connecticut on 10/11/14
For personal use only.

estimate of the fear she may experience during a possible future delivery. Thus an instrument was developed which focused on the specific situation of delivery, with the aim of tracing fear of childbirth by means of the woman's cognitive set (her appraisal in the form of expectancies and experiences) of the delivery of interest. This operationalization of our theoretical construct of fear of childbirth is parallel with Lazarus' theory[6,7], which many theorists more or less agree with[8,9]. Lazarus states that appraisal processes are a principal factor in determining how people react to environmental stressors, and thus also determine the development and maintenance of anxiety.

The development of the Wijma Delivery Expectancy/Experience Questionnaire (W-DEQ version A and B)

The W-DEQ has been developed over the last 10 years. Prototypes have been tested in clinical practice and treatment studies[10]. The intention was to develop a questionnaire which measures fear of childbirth by means of the woman's cognitive appraisal regarding the delivery of interest. The instrument should be appropriate for the measurement in nulliparous as well as parous women, before and after delivery. Equally, the results of the measurements in groups with different parity, as well as from moments pre- and postpartum, should be comparable. Another requirement was that the items had to be concrete as well as directly related to the situation of childbirth.

Thus, the W-DEQ was developed to measure a construct of fear related to childbirth during pregnancy and after delivery by asking the woman about her expectancies before (version A) and experiences after (version B) childbirth, respectively. The content of fear of childbirth was operationalized by means of items comprising statements concerning intensities of emotions and magnitude of cognitions regarding the delivery of present interest. Items were derived from the first two authors' clinical experiences of women with fear of childbirth.

During the development, the questionnaire was tested and patient's remarks concerning convenience and comprehensibility were incorporated. The final form of the W-DEQ discussed here was developed after computations with a 54-item form. After computation of item–total scores, we dropped those items which did not reach a correlation with the total sum ≥ 0.30 in both the nulliparous and parous groups, when measured either in gestation week 32, 2 h after delivery or 5 weeks postpartum. An exception was made for some items which showed a reasonable to good item–total correlation in most situations but not in one (item numbers 10, 21, 23 and 24) or two (item numbers 32 and 33) measurements. Thus 33 items were kept for the final version of the questionnaire, which is presented in Appendices 1 and 2. When filling in the W-DEQ the woman is instructed to rate her personal feelings and cognitions on a six-point Likert scale with the endpoints marked with 'not at all...' and 'extremely...'. A six-point scale was chosen because reliability increases when the number of scale steps increases, but levels off at about seven steps. It was decided to choose six instead of seven scale steps to avoid a response style with neutral responses, which may cause loss of variance[11]. The minimum score is 0, and the maximum score 165. The higher the score, the greater the fear of childbirth manifested, which means that the answers of those questions which are positively formulated (item numbers 2, 3, 6, 7, 8, 11, 12, 15, 19, 20, 24, 25, 27, 31) have to be reversed for the calculation of the woman's individual sum score (see Appendices 1 and 2).

In this paper the questionnaire is presented for the first time, together with item analysis, assessment of reliability (Part 1), and an investigation of the construct validity (Part 2).

PART 1: ITEM ANALYSIS AND RELIABILITY ASSESSMENTS

Methods

Subjects and procedure

Pregnant women, visiting one of the antenatal clinics in Linköping, were invited by their midwives to participate in the study if they met the following inclusion criteria: being in the 28–30th week of gestation, expecting a first, second or third child, and being able to read, understand and speak Swedish. Women who gave their consent were asked to visit the antenatal clinic during their 32nd gestation week to fill in the W-DEQ version A, as well as some other questionnaires (see Part 2). In addition, a copy of the W-DEQ version B was sent to the delivery ward, for the women to fill in within 2 h of delivery. Five weeks postpartum a second

RIGHTSLINK

J Psychosom Obstet Gynaecol Downloaded from informahealthcare.com by University of Connecticut on 10/11/14
For personal use only.

copy of the W-DEQ version B was mailed to all participants. Questionnaires were returned by post.

During a period of 6 months, 196 pregnant women, 96 nulliparous and 100 parous (70 women expecting their second and 30 expecting their third child), completed the W-DEQ version A during gestation week 32. Within 2h after delivery 166 women (78 nulliparous, 88 parous) filled in the W-DEQ version B. (These former nulliparous women were after delivery in fact primiparous. For the sake of convenience the term nulliparous is kept throughout the paper to describe that group which in gestation week 32 was nulliparous.) The women who had a Cesarean section (11 nulliparous, 7 parous) were excluded, because the items in the basic form of the W-DEQ version B were not adjusted to surgical delivery. The remaining 12 women did not fill in the W-DEQ version B 2h after delivery due to tiredness after the delivery or because of organizational mistakes (7 nulliparous, 5 parous). Five weeks postpartum 175 women (84 nulliparous and 91 parous) filled in W-DEQ version B. The women who did not participate in this last measurement were those with a Cesarean section ($n = 18$) and women who for unknown reasons did not return their questionnaire ($n = 3$).

Out of the total of 196 women questioned in gestation week 32, 194 lived with the father of the child. Thirty-four women had an education not beyond nine years of elementary school, 104 had gone through high school, and 58 had a college/university education. The median age of the nulliparous women was 26 years (range 18–39), and of the parous women 29 years (range 19–42). These demographic data suggest that the participating women could be assumed to be repesentative of the population of women giving birth at the Department of Obstetrics and Gynaecology, University Hospital, Linköping[12].

Reliability

Two methods were used for estimating the reliability of the W-DEQ: the internal consistency reliability (Cronbach's α), and the split-half reliability[13]. The split-half method was preferable to the test–retest method, as the expectancy during pregnancy and the appraisal of the experienced delivery is an ongoing psychological process and the woman's cognitive frames of expectancies and experiences are supposed to change when pregnancy is advancing, or when delivery has passed.

Statistics

Item–total correlations of the W-DEQ versions A and B, as well as within group comparisons over moments of measurement were calculated using Pearson's product-moment correlation coefficient. Internal consistency reliability of the W-DEQ versions A and B was estimated by applying the Cronbach-α formula and the Kuder-Richardson formula 20, as described by Carmines and Zeller[13]. Split-half reliability estimates were obtained by dividing the items in an even-numbered and an odd-numbered group of items, after which the Spearman rank correlation with statistical correction was calculated (Spearman–Brown prophecy formula) to obtain the reliability coefficient for the whole test[13].

Results

Item–total analysis

The item scores were correlated with the total sum score for the nulliparous and parous group of women and the three moments of measurement separately. The results are documented in Table 1 and show that, in gestation week 32, the expectations of nulliparous and parous women about the prospective delivery, indicated by the item–total correlations of the W-DEQ, were generally the same, i.e. approximately the same items belonged to the ten highest item–total scores in both groups. The ranking of these ten items was however clearly different in the two groups. The five highest ranked items in the nulliparous group (weak, composed, safe, deserted and desolate) may be associated with weakness and uncertainty, whereas the five highest ranked items in the parous group (lack of self-confidence, panic, frightful, hopelessness, and afraid) more clearly express fear. In the nulliparous group, before delivery, the item–total correlations were also lower than in the parous group.

The ten highest ranked item–total correlations of the measurement 2h after delivery and 5 weeks postpartum show that both groups had become more alike regarding their cognitive appraisal of the (experienced) delivery, as expressed by means of their statements in the W-DEQ version B. The level of the item–total correlations in the nulliparous group became higher immediately after delivery and by the fifth week postpartum had reached the same level as in the parous group in

RIGHTSLINK

J Psychosom Obstet Gynaecol Downloaded from informahealthcare.com by University of Connecticut on 10/11/14
For personal use only.

Table 1 Item–total correlations of the W-DEQ version A and B in nulliparous and parous women in gestation week 32, 2 h after delivery, and 5 weeks after delivery

Item	*Nulliparous group*			*Parous group*		
	W-DEQ A 32nd week gestation ($n = 96$)	*W-DEQ B 2 h after delivery* ($n = 78$)	*W-DEQ B 5 weeks after delivery* ($n = 84$)	*W-DEQ A 32nd week gestation* ($n = 100$)	*W-DEQ B 2 h after delivery* ($n = 88$)	*W-DEQ B 5 weeks after delivery* ($n = 91$)
1. Fantastic	0.41	0.64 (7)	0.56	0.60	0.62	0.70 (10)
2. Frightful	0.37	0.71 (2)	0.81 (2)	0.75 (3)	0.70 (9.5)	0.78 (5)
3. Lonely	0.52	0.44	0.51	0.55	0.43	0.56
4. Strong	0.55 (7)	0.68 (5)	0.75 (4)	0.65	0.80 (1)	0.82 (1)
5. Confident	0.45	0.68 (6)	0.72	0.69 (6)	0.70 (9.5)	0.80 (2)
6. Afraid	0.53 (10)	0.49	0.67 (6)	0.71 (5)	0.66	0.68
7. Deserted	0.58 (4)	0.47	0.65 (9)	0.60	0.48	0.58
8. Weak	0.67 (1)	0.64 (8)	0.73	0.65	0.71 (8)	0.69
9. Safe	0.60 (3)	0.58	0.71	0.66 (9)	0.44	0.61
10. Independent	0.29	0.46	0.63	0.58	0.50	0.71 (7)
11. Desolate	0.57 (5)	0.56	0.63	0.67 (8)	0.74 (5.5)	0.59
12. Tense	0.47	0.55	0.57	0.61	0.74 (5.5)	0.67
13. Glad	0.47	0.49	0.55	0.56	0.67	0.69
14. Proud	0.38	0.51	0.62	0.46	0.68	0.60
15. Abandoned	0.50	0.43	0.63	0.65	0.34	0.49
16. Composed	0.62 (2)	0.70 (3)	0.74 (5)	0.67 (7)	0.72 (7)	0.78 (6)
17. Relaxed	0.55 (9)	0.60	0.67 (7)	0.66 (10)	0.68	0.80 (3)
18. Happy	0.41	0.53	0.60	0.49	0.65	0.64
19. Panic	0.56 (6)	0.63 (10)	0.66 (8)	0.76 (2)	0.78 (3)	0.71 (8)
20. Hopelessness	0.49	0.70 (4)	0.76 (3)	0.74 (4)	0.77 (4)	0.71 (9)
21. Longing for the child	0.31	0.41	0.41	0.40	0.38	0.27
22. Self-confidence	0.55 (8)	0.72 (1)	0.82 (1)	0.80 (1)	0.79 (2)	0.79 (4)
23. Trust	0.36	0.44	0.36	0.50	0.58	0.28
24. Pain	0.27	0.47	0.53	0.58	0.43	0.44
25. Behave badly	0.46	0.46	0.59	0.64	0.65	0.59
26. Let happen	0.42	0.51	0.59	0.54	0.53	0.52
27. Lose control	0.45	0.64 (9)	0.65 (10)	0.55	0.67	0.67
28. Funny	0.42	0.57	0.51	0.60	0.63	0.55
29. Natural	0.50	0.52	0.54	0.44	0.56	0.50
30. Obvious	0.42	0.55	0.60	0.61	0.61	0.45
31. Dangerous	0.48	0.49	0.56	0.64	0.45	0.50
32. Child will die	0.36	0.26	0.48	0.43	0.31	0.24
33. Child will be injured	0.34	0.38	0.50	0.46	0.26	0.22

Figures in parentheses indicate the ranking of the ten items with the highest item–total correlations per moment of measurement; W-DEQ, Wijma Delivery Expectancy/Experience Questionnaire

gestation week 32. (For all item–total correlations in versions A and B, $p < 0.0001$).

Pre- and postpartum comparisons

The equivalence of the W-DEQ for comparing antepartum and postpartum scores is shown in Table 2. Over time, the Pearson correlation coefficients are exactly the same for the total sample as for both groups separately ($p < 0.0001$). This means that, in the nulliparous as well as in the parous women, the W-DEQ ordered the women within the respective groups in exactly the same way at the different moments of measurement.

RIGHTSLINK

Table 2 Pearson correlation coefficients in a group of nulliparous and parous women between W-DEQ scores in 32nd gestation week, 2h after delivery and 5 weeks after delivery

	Total sample	*Nulliparous group*	*Parous group*
w 32 × 2 h	0.64	0.64	0.64
w 32 × 5 w	0.64	0.60	0.67
2 h × 5 w	0.83	0.84	0.83

All correlations $p < 0.0001$; w 32, 32nd week of gestation; 2 h, 2h after delivery; 5 w, 5 weeks postpartum

Reliability

Reliability estimations for the W-DEQ versions A and B are presented in Table 3. Both the split-half correlation coefficients and the alpha coefficients are very high. All but two coefficients (nulliparous group, gestation week 32) are higher than 0.90.

PART 2: CONSTRUCT VALIDITY OF THE W-DEQ

Methods

Measures

The analysis of the W-DEQ version A was continued with an investigation of its construct validity. (Construct validity refers to the extent to which a questionnaire measures a theoretical construct or trait.) The questionnaires described below were, together with the W-DEQ version A, administered in gestation week 32 to the same group of pregnant women as in Part 1 (96 nulliparous women and 100 parous women).

The S-R Inventory of Anxiousness (SRI)[14] This scale measures the experienced intensity of 14 physiological correlates of anxiety while imagining a fearful situation, in this case the state during delivery when labor is intensive and the cervix is dilated to six centimetres.

The Fear Questionnaire (FQ) The FQ used in this study is the first scale from Marks and Mathews' Fear Questionnaire[15], which measures phobic fears in different situations. Items regarding fear of childbirth and the gynecological examination were added to the original list of anxiety provoking situations, making 20 items in total.

The State–Trait Anxiety Inventory (STAI)[16] The STAI measures trait and state anxiety. The version of STAI used in this study is the part measuring trait anxiety (20 items). The women were instructed to refer to the present situation of the pregnancy.

The Karolinska Scales of Personality (KSP) This measures stable personality traits. In this study only subscales measuring psychic anxiety, somatic anxiety and muscular tension were used, totalling 30 items[17,18].

The Eysenck Personality Inventory (EPI)[19–21] The EPI consists of 57 items and measures personality dimensions of neuroticism (EPI-N) and extraversion (EPI-E).

The Internal–External Locus of Control Scale (I–E)[22] The IE, as used in this study, is a version adjusted to a pregnant population. The scale (22 items) is thought to measure the degree to which the woman believes that those good and bad events that happen to her are under her own control (low score) or under the control of external factors such as chance or fate (high score).

The Beck Depression Inventory (BDI) The BDI measures severity of depressive states[23,24]. Eight questions about physiological reactions were removed because in a pregnant population these reactions may be caused by the pregnancy instead of being symptoms of depression, thus resulting in a 13-item version.

To test the construct of fear of childbirth the SRI and the FQ-childbirth, both directly related to childbirth, were included. To test if the W-DEQ measured in the domain of anxiety, the STAI, KSP and the EPI were added. The trait version of the STAI was chosen. Compared to the situation before, pregnancy brings a great psychological change in many women's lives. Therefore the STAI instruction was adjusted to include the present situation of pregnancy. The trait version was chosen to compare the content of the W-DEQ with a general trait, avoiding accidental changes which would be measured by means of the state version. To further study the construct of the W-DEQ, the series was completed with IE and BDI, because

J Psychosom Obstet Gynaecol Downloaded from informahealthcare.com by University of Connecticut on 10/11/14
For personal use only.

RIGHTSLINK

Table 3 Reliability estimates of nine questionnaires in nulliparous and parous women. Split-half (after correction with the Spearman–Brown prophecy formula), Cronbach's alpha, and Kuder-Richardson reliability estimates of W-DEQ version A (before delivery), SRI, Fear Questionnaire, STAI, KSP, EPI-N, EPI-E, I-E, BDI, and W-DEQ version B (2 h and 5 weeks postpartum) in nulliparous and parous women

	Combined groups	*Nulliparous group*	*Parous group*
W-DEQ			
Version A, pregnancy week 32			
Split-half reliability	1.00	0.87	0.96
Cronbach's alpha	0.93	0.89	0.99
	(*n* = 196)	(*n* = 96)	(*n* = 100)
Version B, 2 h after delivery			
Split-half reliability	0.95	0.92	0.96
Cronbach's alpha	0.93	0.92	0.94
	(*n* = 166)	(*n* = 78)	(*n* = 88)
Version B, 5 weeks after delivery			
Split-half reliability	0.96	0.92	0.96
Cronbach's alpha	0.94	0.94	0.94
	(*n* = 175)	(*n* = 84)	(*n* = 91)
SRI, pregnancy week 32			
Cronbach's alpha	0.82	0.86	0.87
	(*n* = 196)	(*n* = 96)	(*n* = 100)
FQ, pregnancy week 32			
Cronbach's alpha	0.83	0.88	0.84
	(*n* = 196)	(*n* = 96)	(*n* = 100)
STAI, pregnancy week 32			
Cronbach's alpha	0.88	0.90	0.92
	(*n* = 196)	(*n* = 96)	(*n* = 100)
KSP, pregnancy week 32			
Cronbach's alpha	0.89	0.92	0.91
	(*n* = 196)	(*n* = 96)	(*n* = 100)
EPI-N, pregnancy week 32			
Kuder-Richardson	0.76	0.76	0.76
	(*n* = 196)	(*n* = 96)	(*n* = 100)
EPI-E, pregnancy week 32			
Kuder-Richardson	0.52	0.52	0.58
	(*n* = 196)	(*n* = 96)	(*n* = 100)
I-E, pregnancy week 32			
Kuder-Richardson	0.63	0.56	0.65
	(*n* = 196)	(*n* = 96)	(*n* = 100)
BDI, pregnancy week 32			
Cronbach's alpha	0.71	0.74	0.78
	(*n* = 150)	(*n* = 83)	(*n* = 67)

W-DEQ, Wijma Delivery Expectancy/Experience Questionnaire; SRI, S-R Inventory of Anxiousness; FQ, Fear Questionnaire (childbirth, agora, social, injury, gynecological examination, elevator, darkness); STAI, Spielberger Trait Anxiety Inventory; KSP, Karolinska Scales of Personality; EPI-N, Neuroticism scale of Eysenck's Personality Inventory; EPI-E, Extraversion scale of Eysenck's Personality Inventory; I-E, Internal–External Locus of Control Scale; BDI, Beck's Depression Inventory

J Psychosom Obstet Gynaecol Downloaded from informahealthcare.com by University of Connecticut on 10/11/14
For personal use only.

RIGHTSLINK

J Psychosom Obstet Gynaecol Downloaded from informahealthcare.com by University of Connecticut on 10/11/14
For personal use only.

these scales could be expected to correlate inversely with the W-DEQ.

Analysis of construct validity

Analysis of the construct validity of the W-DEQ version A by means of correlation with the scales mentioned above (Pearson's product–moment correlation coefficients)[11] was performed for both nulliparous and parous women separately. It could be expected that the W-DEQ would have the highest correlations with FQ-childbirth and SRI. Because the relationship between different forms of anxiety is well known[25], it could be expected that the W-DEQ would have more overlap with the questionnaires measuring general and specific anxiety (STAI, KSP, FQ except childbirth, EPI-N) than with the questionnaires measuring depression (BDI) and internal–external locus of control (I–E), whereas extraversion (EPI-E) could be expected not at all to relate to the content of the W-DEQ. Depending on Eysenck's description of neuroticism as 'a personality variable reflecting autonomic reactivity, a propensity to respond to stressors by developing anxiety'[19,20], the EPI-N scale was reckoned among the questionnaires measuring anxiety. Thus, regarding the expected correlations between the W-DEQ and the other questionnaires the following was hypothesized:

(W-DEQ × FQ-childbirth; W-DEQ × SRI) > (W-DEQ × STAI; W-DEQ × KSP; W-DEQ × FQ except childbirth; W-DEQ × EPI-N) > (W-DEQ × BDI; W-DEQ × I-E) > (W-DEQ × EPI-E)

Because it was the intention that the W-DEQ would measure childbirth-related anxiety in both the nulliparous as well as the parous group at the different times of measurement, it was expected that no difference would appear between the two groups, neither regarding the correlations between the W-DEQ and FQ-childbirth/SRI, nor between the W-DEQ and the rest of the questionnaires.

Statistics

Internal consistency reliability of the questionnaires other than W-DEQ versions A and B was estimated by applying the Cronbach-α formula and the Kuder-Richardson formula 20[13]. Correlations were computed by means of Pearson's product– moment correlation coefficients. The test of the same true value of correlations between the W-DEQ and the other questionnaires in the two groups was performed according to Hays[26]. For testing the significance of the 28 correlations of the W-DEQ version A with the other questionnaires, only significance levels of 0.001 or smaller were accepted, in order to reduce the risk of Type 1 error.

Results

The reliability estimations of all questionnaires are presented in Table 3, and construct validity measures are shown in Table 4. For the nulliparous group, the hypothesis that the W-DEQ would correlate highest with both FQ-childbirth and the SRI was not confirmed. Correlations between the W-DEQ and these specific measurements were of the same magnitude as those with the questionnaires measuring anxiety in general. Of the other questionnaires, only FQ-social fear correlated significantly with the W-DEQ. The remaining correlations were not statistically significant. On the other hand, in the parous group, the first part of the hypothesis was generally confirmed. However, the correlation of the W-DEQ with the BDI was shown to be as high as with the anxiety scales. A further analysis showed that the BDI also correlated nearly on the same level with the other scales measuring general anxiety (STAI, 0.71; KSP, 0.58; EPI-N, 0.55; all correlations $p < 0.0001$). The remaining correlations were not statistically significant.

Table 4 shows that the hypothesis that the correlation of the W-DEQ with the other questionnaires would be the same for both the nulliparous and parous group could be confirmed, except for FQ-childbirth.

After the findings of the differences between the two groups, additional analyses were performed. Because the two groups differed only regarding experience with delivery, it was hypothesized that the SRI and the FQ-childbirth would correlate in the same way with the other questionnaires as the W-DEQ, as well as that correlations between the questionnaires that were not especially related to delivery would be the same in both groups. (Additional correlations were only computed for those questionnaires that showed significant correlations in Table 4.) Table 5 shows that in most cases the hypothesis was confirmed. Especially the STAI and the KSP, which had good reliability (> 0.90,

RIGHTSLINK

J Psychosom Obstet Gynaecol Downloaded from informahealthcare.com by University of Connecticut on 10/11/14
For personal use only.

Table 4 Pearson's correlations (r) and their 95% confidence intervals (CI) between W-DEQ version A and other questionnaires in a group of nulliparous ($n = 96$; BDI, $n = 67$) and parous ($n = 100$; BDI, $n = 83$) women during their 32nd week of pregnancy

	W-DEQ nulliparous group		*W-DEQ parous group*			
	r_1	*95% CI*	r_2	*95% CI*	Zr_1-r_2	*p*
SRI	0.52** (2)	0.36–0.65	0.65** (2)	0.51–0.75	1.37	NS
FQ: -childbirth	0.43** (3.5)	0.25–0.60	0.78** (1)	0.69–0.85	4.04	0.0001
-agora	0.27	0.08–0.45	0.33* (7)	0.14–0.49	0.46	NS
-social	0.34* (6)	0.14–0.50	0.44** (6)	0.27–0.59	0.81	NS
-injury	0.17	0.04–0.36	0.28	0.09–0.45	0.80	NS
-gyn. ex.	0.26	0.06–0.43	0.32* (8)	0.14–0.49	0.45	NS
-elevator	0.16	–0.04–0.35	0.20	0.00–0.38	0.29	NS
-darkness	0.07	–0.13–0.27	0.23	0.03–0.41	1.13	NS
STAI	0.54** (1)	0.38–0.67	0.55** (3)	0.40–0.68	0.10	NS
KSP	0.43** (3.5)	0.25–0.58	0.47** (4.5)	0.31–0.61	0.35	NS
EPI-N	0.38** (5)	0.19–0.54	0.38** (9)	0.20–0.54	0.00	NS
EPI-E	0.10	–0.10–0.30	0.03	–0.22–0.17	–0.49	NS
I-E	0.10	–0.11–0.29	0.28	0.09–0.45	1.29	NS
BDI	0.26	0.02–0.47	0.47** (4.5)	0.29–0.63	1.46	NS

*$p \leq 0.001$; **$p \leq 0.0001$; Zr_1-r_2, test of difference between correlations in two groups[25]; figure within parenthesis is the coefficient order; NS, not significant; W-DEQ, Wijma Delivery Expectancy/Experience Questionnaire; SRI, S-R Inventory of Anxiousness; FQ, Fear Questionnaire (childbirth, agora, social, injury, gynecological examination, elevator, darkness); STAI, Spielberger Trait Anxiety Inventory; KSP, Karolinska Scales of Personality; EPI-N, Neuroticism scale of Eysenck's Personality Inventory; EPI-E, Extraversion scale of Eysenck's Personality Inventory; I-E, Internal–External Locus of Control Scale; BDI, Beck's Depression Inventory

Table 3), showed in both groups almost exactly similar correlations with the other questionnaires.

DISCUSSION

When constructing the W-DEQ, the intention was to develop a pool of items which together measure the construct of fear of childbirth, both in nulliparous and parous pregnant women. However, the item–total correlations from pregnancy week 32 showed that the construct of the W-DEQ for the nulliparous group not only comprised fear related to childbirth but also uncertainty. This difference between the two groups might be explained by the fact that it is easier for parous pregnant women to imagine the situation of childbirth. As a confirmation of this assumption, after the delivery, the items that appeared to dominate the construct were the same in both groups.

In attempting to develop a reliable, concrete and easily comprehensible instrument, we appear to have had success. Both in nulliparous and parous women, as well as at the three different moments of measurements, the W-DEQ appears to have a high reliability as estimated by means of split-half coefficients and coefficients alpha, even meeting Nunnally's norm of 0.90–0.95 for instruments in applied settings[11], although the W-DEQ at present only is a research instrument and therefore an alpha coefficient of about 0.70 would have been sufficient.

When testing if a new questionnaire measures a specific part of anxiety, one has to balance between accepting a reasonable part of overlap with other anxiety questionnaires, to prove that the questionnaire measures within the domain of anxiety, and avoiding too large an overlap, which would make the new questionnaire redundant. The W-DEQ correlated respectively 0.54 (nulliparous group) and 0.55 (parous group) with the STAI, meaning 30% overlap. Because both the W-DEQ and the STAI showed high reliability (> 0.90), most of this overlap may be seen as common true variance. This level of common variance indicates that the W-DEQ measures in the domain of anxiety, and, at the same time, that enough variance is left for the measurement of another dimension.

In the parous group the W-DEQ correlated 0.78 and 0.65 with the SRI and FQ-childbirth,

RIGHTSLINK

J Psychosom Obstet Gynaecol Downloaded from informahealthcare.com by University of Connecticut on 10/11/14
For personal use only.

Table 5 Intercorrelations in the nulliparous and parous groups between the questionnaires measuring fear of childbirth and other questionnaires

	W-DEQ A week 32		*SRI*		*FQ-child*		*FQ-agora*		*FQ-social*		*FQ-gyn. ex.*		*EPI-N*		*KSP*		*STAI*		*BDI*		*W-DEQ B 2h*	
	np	*p*	*np*	*p*	*np*	*p*	*np*	*p*	*np*	*p*	*np*	*p*	*np*	*p*	*np*	*p*	*np*	*p*	*np*	*p*	*np*	*p*
SRI	0.52	0.65																				
FQ-childbirth	0.43	0.78	0.43	0.58																		
FQ-agora	0.27	0.33	0.27	0.15	0.36	0.31																
FQ-social	0.34	0.44	0.36	0.38	0.46	0.45	0.61	0.56														
FQ-gyn. ex.	0.26	0.32	0.26	0.31	0.47	0.40	0.43	0.35	0.38	0.36												
EPI-N	0.38	0.38	0.38	0.36	0.40	0.36	0.39	0.44	0.58	0.40	0.30	0.25										
KSP	0.43	0.47	0.43	0.46	0.42	0.35	0.45	0.53	0.58	0.47	0.26	0.25	0.66	0.71								
STAI	0.54	0.55	0.54	0.46	0.36	0.40	0.36	0.33	0.46	0.41	0.26	0.26	0.70	0.65	0.76	0.74						
BDI	0.26	0.47	0.05	0.40	0.22	0.36	0.43	0.24	0.50	0.47	0.45	0.47	0.55	0.55	0.50	0.58	0.55	0.71				
W-DEQ B 2h	0.64	0.64	0.33	0.56	0.33	0.43	0.10	0.33	0.18	0.25	0.26	0.32	0.29	0.39	0.35	0.47	0.45	0.45	0.33	0.41		
W-DEQ B 5 weeks	0.60	0.67	0.33	0.59	0.24	0.52	0.16	0.30	0.27	0.33	0.31	0.27	0.42	0.41	0.46	0.46	0.48	0.44	0.40	0.49	0.84	0.83

np, nulliparous group; p, parous group; W-DEQ, Wijma Delivery Expectancy/Experience Questionnaire; SRI, S-R Inventory of Anxiousness; FQ, Fear Questionnaire (childbirth, agora, social, gynecological examination); STAI, Spielberger Trait Anxiety Inventory; KSP, Karolinska Scales of Personality; EPI-N, Neuroticism scale of Eysenck's Personality Inventory; BDI, Beck's Depression Inventory

RIGHTSLINK

J Psychosom Obstet Gynaecol Downloaded from informahealthcare.com by University of Connecticut on 10/11/14
For personal use only.

respectively, in other words an overlap of 61% and 42%, indicating, in accordance with the hypothesis, that the common variance is higher for the W-DEQ and these questionnaires than for the W-DEQ and those questionnaires measuring general anxiety. This means that the W-DEQ also measures aspects related to direct communication of fear of childbirth (FQ-childbirth) and physiological symptoms related to fear when imagining labor and delivery (SRI). Unfortunately, this hypothesis could not be affirmed for the nulliparous group. To study the difference between the two groups, a further analysis was performed. Except for the correlation between the SRI and the BDI, no other systematic difference between the two groups appeared, indicating that the differences found, in all probability, had to do with the lack of experience of labor and delivery of the nulliparous group.

According to the results mentioned above, directly after the delivery the item–total correlations of the two groups became more alike. This change was in spite of the difference in how the two groups reacted to the specific items of the W-DEQ in pregnancy week 32. This may show that the W-DEQ before and after delivery in the nulliparous group has the same potency to select women with different kinds of cognitive appraisals regarding childbirth as in the parous group.

The W-DEQ is presented here for the first time; however, further research, especially replications of the psychometric work, in comparable samples is needed. Further research with the W-DEQ should concern predictive validity (to predict pregnant women's status at labor and after delivery), concurrent validity (to be able to use the W-DEQ for assessment and diagnostic aims in individual patients) and computations of norm values. This would allow the W-DEQ to be used as both a research instrument and a questionnaire for use in clinical practice. Recent research has shown that the predictive validity of the W-DEQ is promising[10], and that the W-DEQ can be helpful in interpreting post-traumatic stress disorder after childbirth[12].

ACKNOWLEDGEMENTS

The authors thank Ing-Marie Björnstedt for her assistance in collecting the data, and Dr Jan Hosman for engaged discussions about the psychometric analyses.

REFERENCES

1. Istvan J. Stress, anxiety, and birth outcomes: a critical review of the evidence. *Psychol Bull* 1986;100: 331–48
2. Levin JS, DeFrank R. Maternal stress and pregnancy outcomes: a review of the psychosocial literature. *J Psychosom Obstet Gynecol* 1988;9:3–16
3. Lobel M. Conceptualizations, measurement, and effects of prenatal maternal stress on birth outcomes. *J Behav Med* 1994;17:225–72
4. Reading AE. The influence of maternal anxiety on the course and outcome of pregnancy: a review. *Health Psych* 1983;2:187–202
5. Areskog B, Kjessler B, Uddenberg N. Identification of women with significant fear of childbirth during late pregnancy. *Gynecol Obstet Invest* 1982;13:98–107
6. Lazarus RS. Thoughts on the relations between emotion and cognition. *Am Psychol* 1982;37:1019–24
7. Lazarus RS, Averill JR. Emotion and cognition: with special reference to anxiety. In: Spielberger CD, ed. *Anxiety: Current Trends in Theory and Research*, vol 2. New York: Academic Press 1972;241–83
8. Edelmann RJ. *Anxiety: Theory, Research and Intervention in Clinical and Health Psychology*. Chichester: John Wiley and Sons 1992
9. Eysenck MW. *Anxiety: the Cognitive Perspective*. Hove: Lawrence Erlbaum Associates 1992
10. Wijma K, Wijma B. Changes in anxiety during pregnancy and after delivery. In Wijma K, Von Schoultz B, eds. *Reproductive Life*. London: The Parthenon Publishing Group 1992;81–88
11. Nunnally JC. *Psychometric Theory*. New York: McGraw Hill, 1967
12. Wijma K, Söderquist J, Wijma B. Post-traumatic stress disorder after childbirth. A cross sectional study. *J Anx Dis* 1998, in press
13. Carmines EG, Zeller RA. *Reliability and validity assessent*. Sage University Paper Series on Quantative Applications in the Social Sciences, series no. 07-017. Beverly Hills: Sage Publications 1988
14. Endler NS, Hunt J, Rosenstein AJ. An S-R inventory of anxiousness. *Psych Mon* 1962;76:1–33
15. Marks IM, Mathews AM. Brief standard self-rating for phobic patients. *Behav Res Ther* 1979;17:263–7
16. Spielberger CD, Gorsuch RL, Lushene R, *et al. Manual for the State-Trait Anxiety Inventory*. Palo Alto: Consulting Psychologists Press 1983
17. AF Klinteberg B, Schalling D, Magnusson D. *Self-Report Assessment of Personality Traits*. Reports from the Department of Psychology no 64. Stockholm:

RIGHTSLINK

University of Stockholm, Department of Psychology 1986

18. Von Knorring L, Von Knorring A-L, Smigan L *et al.* Personality traits in subtypes of alcoholics. *J Stud Alcoh* 1987;48:523–7
19. Eysenck HJ, Eysenck SBG. *Manual of the Eysenck Personality Inventory.* London: University of London Press 1964
20. Eysenck HJ, Eysenck SBG. *The Manual of the Eysenck Personality Questionnaire*. Sevenoaks: Hodder and Stoughton, 1975
21. Bederoff-Petersson A, Jägtoft K, Åström J. *EPI Eysenck Personality Inventory: Synpunkter och några svenska undersökningsdata.* Stockholm: Psykologi Förlaget 1968
22. Rotter JB. Generalized expectations for internal versus external control of reinforcement. *Psychol Mon* 1966;80:1–28
23. Beck AT. *Depression: Causes and Treatment.* Philadelphia: University of Pennsylvania Press 1967
24. Jansson L. *Handbok i kognitiv terapi vid depression.* Stockholm: Natur och Kultur, 1986
25. Öhman A. Fear and anxiety as emotional phenomenon: clinical phenomenology, evolutionary perspectives, and information processing mechanisms. In Lewis M, Haviland JM, eds. *Handbook of Emotions.* New York: Guilford Publications 1993;511–36
26. Hays WL. *Statistics,* 3rd edn. New York: Holt Saunders 1981
27. Ryding EL, Wijma K, Wijma B, *et al.* Fear of chilbirth during pregnancy may increase the risk of emergency Cesarean section. *Acta Obstet Gynecol Scand* 1998;in press

Received 6 January 1997; accepted 15 August 1997

APPENDIX 1 The Wijma Delivery Expectancy/Experience Questionnaire (W-DEQ) version A

© 1996 K. Wijma & B. Wijma

Instruction

This questionnaire is about feelings and thoughts women may have at the prospect of labour and delivery.

The answers to each question appear as a scale from 1 to 6. The outermost answers (1 and 6 respectively) correspond to the opposite extremes of a certain feeling or thought.

Please complete each question by drawing a circle around the number belonging to the answer which most closely corresponds to **how you imagine** your labour and delivery will be.

Please answer **how you imagine** your labour and delivery will be – not the way you hope it will be.

I How do you think your labour and delivery will turn out as a whole?

	1	2	3	4	5	6
1	Extremely fantastic					Not at all fantastic
2	Extremely frightful					Not at all frightful

II How do you think you will feel in general during the labour and delivery?

	1	2	3	4	5	6
3	Extremely lonely					Not at all lonely
4	Extremely strong					Not at all strong
5	Extremely confident					Not at all confident
6	Extremely afraid					Not at all afraid
7	Extremely deserted					Not at all deserted
8	Extremely weak					Not at all weak
9	Extremely safe					Not at all safe
10	Extremely independent					Not at all independent
11	Extremely desolate					Not at all desolate

J Psychosom Obstet Gynaecol Downloaded from informahealthcare.com by University of Connecticut on 10/11/14
For personal use only.

12 1 2 3 4 5 6
Extremely tense — Not at all tense

13 1 2 3 4 5 6
Extremely glad — Not at all glad

14 1 2 3 4 5 6
Extremely proud — Not at all proud

15 1 2 3 4 5 6
Extremely abandoned — Not at all abandoned

16 1 2 3 4 5 6
Totally composed — Not at all composed

17 1 2 3 4 5 6
Extremely relaxed — Not at all relaxed

18 1 2 3 4 5 6
Extremely happy — Not at all happy

III How do you think you will feel during the labour and delivery?

19 1 2 3 4 5 6
Extreme panic — No panic at all

20 1 2 3 4 5 6
Extreme hopelessness — No hopelessness at all

21 1 2 3 4 5 6
Extreme longing for the child — No longing for the child at all

22 1 2 3 4 5 6
Extreme self-confidence — No self-confidence at all

23 1 2 3 4 5 6
Extreme trust — No trust at all

24 1 2 3 4 5 6
Extreme pain — No pain at all

IV What do you think will happen when labour is most intense?

25 1 2 3 4 5 6
I will behave extremely badly — I will not behave badly at all

26 1 2 3 4 5 6
I will dare to totally surrender control to my body — I will not dare to surrender control to my body at all

27 1 2 3 4 5 6
I will totally lose control of myself — I will not lose control of myself at all

V How do you imagine it will feel the very moment you deliver the baby?

28 1 2 3 4 5 6
Extremely funny — Not at all funny

29 1 2 3 4 5 6
Extremely natural — Not at all natural

30 1 2 3 4 5 6
Extremely self-evident — Not at all self-evident

31 1 2 3 4 5 6
Extremely dangerous — Not at all dangerous

VI Have you, during the last month, had fantasies about the labour and delivery, for example…

32 …fantasies that your child will die during labour/delivery?
1 2 3 4 5 6
Never — Very often

33 …fantasies that your child will be injured during labour/delivery?
1 2 3 4 5 6
Never — Very often

Would you please now check that you have not forgotten to answer any questions.

J Psychosom Obstet Gynaecol Downloaded from informahealthcare.com by University of Connecticut on 10/11/14
For personal use only.

J Psychosom Obstet Gynaecol Downloaded from informahealthcare.com by University of Connecticut on 10/11/14
For personal use only.

APPENDIX 2 The Wijma Delivery Expectancy/Experience Questionnaire (W-DEQ) version B

© 1996 K. Wijma & B. Wijma

Instruction

This questionnaire is about feelings and thoughts women may have after childbirth.

The answers to each question appear as a scale from 1 to 6. The outermost answers (1 and 6 respectively) correspond to the opposite extremes of a certain feeling or thought.

Please complete each question by drawing a circle around the number belonging to the answer which most closely corresponds to **how you now think** your labour and delivery was.

Please answer **how you now think** your delivery was – not the way you wish it would have been.

I How did you experience your labour and delivery as a whole?

	1	2	3	4	5	6
1	Extremely fantastic					Not at all fantastic
2	Extremely frightful					Not at all frightful

II How did you feel in general during the labour and delivery?

	1	2	3	4	5	6
3	Extremely lonely					Not at all lonely
4	Extremely strong					Not at all strong
5	Extremely confident					Not at all confident
6	Extremely afraid					Not at all afraid
7	Extremely deserted					Not at all deserted
8	Extremely weak					Not at all weak
9	Extremely safe					Not at all safe
10	Extremely independent					Not at all independent
11	Extremely desolate					Not at all desolate
12	Extremely tense					Not at all tense
13	Extremely glad					Not at all glad
14	Extremely proud					Not at all proud
15	Extremely abandoned					Not at all abandoned
16	Extremely composed					Not at all composed
17	Extremely relaxed					Not at all relaxed
18	Extremely happy					Not at all happy

III What did you feel during the labour and delivery?

	1	2	3	4	5	6
19	Extreme panic					No panic at all
20	Extreme hopelessness					No hopeless-ness at all
21	Extreme longing for the child					No longing for the child at all
22	Extreme self-confidence					No self-confidence at all
23	Extreme trust					No trust at all
24	Extreme pain					No pain at all

IV What happened when the labour was most intense?

	1	2	3	4	5	6
25	I behaved extremely badly					I did not behave badly at all

J Psychosom Obstet Gynaecol Downloaded from informahealthcare.com by University of Connecticut on 10/11/14
For personal use only.

26 1 2 3 4 5 6
I dared to totally surrender control to my body — I did not dare surrender control to my body at all

27 1 2 3 4 5 6
I lost total control of myself — I did not lose control of myself at all

V How was the very moment you delivered the baby?

28 1 2 3 4 5 6
Extremely funny — Not at all funny

29 1 2 3 4 5 6
Extremely natural — Not at all natural

30 1 2 3 4 5 6
Extremely self-evident — Not at all self-evident

31 1 2 3 4 5 6
Extremely dangerous — Not at all dangerous

VI Had you, during the labour and delivery, fantasies like for example…

32 …fantasies that your child would die during labour/delivery?
1 2 3 4 5 6
Never — Very often

33 …fantasies that your child would be injured during labour/delivery?
1 2 3 4 5 6
Never — Very often

Would you please now check that you have not forgotten to answer any questions.

RIGHTSLINK
